# Employing Bidirectional Two-Sample Mendelian Randomization Analysis to Verify the Potential of Polyunsaturated Fatty Acid Levels in the Prevention of Pancreatic Cancer

**DOI:** 10.3390/cimb46060360

**Published:** 2024-06-14

**Authors:** Hao Sha, Weifeng Zhu

**Affiliations:** School of Basic Medical Sciences, Jiangxi Medical College, Nanchang University, Nangchang 330006, China; sh1277442661@gmail.com

**Keywords:** fatty acids, Omega-3, Omega-6, pancreatic cancer, Mendelian randomization, GWAS

## Abstract

Polyunsaturated fatty acids (PUFAs), specifically Omega-3 (FAω3) and docosahexaenoic acid (DHA), have been studied for their potential role in modulating pancreatic cancer (PC) risk. Although observational studies suggest a beneficial effect in reducing this risk, their findings are often limited by confounding variables and issues of reverse causation. This study used a two-way two-sample Mendelian randomization (MR) method to test the hypothesized genetic causal relationship between PUFAs and PC risk. Data from an extensive genome-wide association study (GWAS) were analyzed, focusing on FAω3 and FAω6 levels, their ratios, and DHA as variables and PC incidence as outcomes. This relationship was comprehensively evaluated using related MR methods, such as inverse variance weighting (IVW), MR Egger, and weighted median (WM). This study finds a significant negative correlation between FAω3 and DHA levels and PC risk, while FAω6 levels show no significant correlation. Interestingly, the ratio of FAω6 to FAω3 was positively associated with increased risk of PC. Neither the MR Egger nor the MR-PRESSO tests detected significant pleiotropy, nor did the Cochrane’s Q test show significant heterogeneity. Leave-one-out analyzes further confirmed the robustness of these results. Using MR analysis of two samples, this study provides genetic causal evidence that FAω3 and DHA levels reduce the risk of PC, whereas the ratio of FAω6 to FAω3 increases the risk of PC. These insights highlight the potential utility of supplementing FAω3 and DHA or altering PUFAs in developing PC prevention strategies.

## 1. Introduction

Polyunsaturated fatty acids (PUFAs) are widely present in diets globally, exerting significant influence on human health [1]. PUFAs are categorized into FAω3 and FAω6 fatty acids, which are essential nutrients that the human body cannot synthesize; therefore, they must be acquired through dietary sources [2]. The consumption levels and dietary ratios of PUFAs vary significantly across different regions, reflecting diverse dietary practices. Numerous observational studies have linked adequate intake of PUFAs to a spectrum of health benefits, such as reduced cardiovascular disease risk, enhanced cognitive function, and decreased inflammation [3,4]. Nonetheless, disease-specific studies are often affected by confounding factors that complicate establishing a causal relationship [5]. In particular, research investigating the associations between PUFAs and specific diseases has produced inconsistent findings [6]. Recognizing and understanding these disparities are vital for tailoring personalized prevention and therapeutic strategies.

Pancreatic cancer (PC) ranks among the most aggressive and lethal malignancies worldwide, with the survival rate remaining largely stagnant over recent decades and a five-year survival rate not exceeding 10% [7]. The prevalence of PC exhibits considerable variation based on geographical location and lifestyle factors, with incidence rates fluctuating between 10% and 25% across different demographics [8]. This disease is characterized by high postoperative complications, scarce therapeutic options, disappointing treatment outcomes, and a profound deterioration in patient quality of life. The frequently asymptomatic progression of PC in its early stages, coupled with the challenges of early detection using contemporary medical technologies, underscores the critical need for research focused on identifying modifiable risk factors [9]. Current investigations into the relationship between PUFAs and PC primarily adopt a broad research approach [10,11]. There is a pressing need for future research to meticulously explore the nuanced segmentation of disease stages. Such an in-depth approach will enable healthcare practitioners to devise more tailored interventions, acknowledging the complexity of PUFA-disease relationships and enhancing the prevention and treatment protocols for PC [12,13].

Mendelian Randomization (MR) studies leverage genetic variants as instrumental variables (IVs) to estimate the causal influence of exposures (e.g., lifestyle and environmental factors, or disease status) on health outcomes, circumventing the biases associated with confounding factors. This methodology rests on the principle that genetic variations are fixed early in an individual’s life and are distributed in a manner analogous to the random assignment in randomized controlled trials [14]. Consequently, by utilizing genetic variants as proxies for exposure levels, MR Studies can significantly mitigate the effects of confounders prevalent in conventional observational studies, offering a more reliable basis for inferring causal relationships [15]. In the current research, we apply a two-sample MR analysis, drawing on summary data from GWAS, to investigate the potential causal link between specific PUFAs and PC. This approach enables the use of extensive genetic information to elucidate the causal pathways between these variables, thus furnishing scientific evidence that can inform future preventive and therapeutic strategies.

## 2. Materials and Methods

### 2.1. Study Design

**Figure 1:**
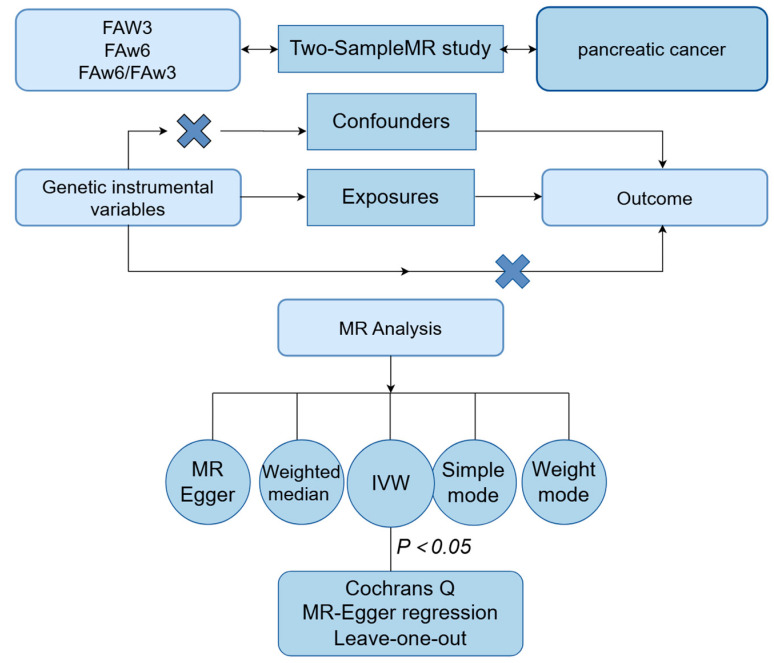
Created using Figdraw. Illustration of MR studies elucidating the causal relationship between PUFAs and PC. MR: Mendelian Randomization; FAω3, Omega-3 fatty acids levels; FAω6, Omega-6 fatty acids levels; FAω6/FAω3, Omega-6 to Omega-3 ratio levels; PC, pancreatic cancer. The error symbol indicates no correlation.

This research employs bidirectional two-sample MR analyses to investigate the causal dynamics between levels of various PUFAs and the risk of PC. Unlike single-sample MR, the two-sample MR approach amalgamates data from comprehensive GWAS, utilizing SNVs (single nucleotide variants) as IVs for enhanced efficacy and robustness [16]. In this investigation, bidirectional MR analyses assess the genetic correlations between PUFA-related genetic variants—including FAω3 levels, DHA within the Omega-3 family, FAω6 levels, and the FAω6 to FAω3 ratio—and PC risk. A conceptual framework for this two-sample MR methodology is outlined and visualized in Figure 1, facilitating a comprehensive exploration of the interplay between PUFAs and PC susceptibility.

### 2.2. Data Sources

This investigation utilizes the latest genetic summary data from the UK Biobank in Europe (https://ukbiobank.ac.uk/, accessed on 6 March 2024) for both exposure and outcome variables. The PUFAs research is anchored in a broad-scale population cohort comprising approximately 500,000 participants, representing about 5% of the total population invited to participate [17]. The GWAS for PUFAs encompasses 115,006 samples, while the PC summary statistics include 1196 cases and 475,049 controls. All participants were verified to be of European descent.

### 2.3. Selection of Instrumental Variables

Prior to executing MR analyses, we rigorously adhered to the three foundational assumptions of MR to bolster the precision of causal inferences:

(1) Relevance Assumption Genetic variants must exhibit a strong association with the exposure, confirmed through genome-wide significance (*p* < 5 × 10^−8^).

(2) Independence Assumption: To counteract potential biases from strong linkage disequilibrium (LD) among selected SNVs, we ensured no LD clustering among the IVs. “Clumping” was employed to select independent SNVs (R^2^ < 0.001, physical distance = 10,000 kb), maintaining IVs’ independence [18].

(3) Exclusion Assumption: The comparison of effect allele types and frequencies facilitated the alignment of SNVs related to the outcome with those associated with the exposure. SNVs with palindromic alleles were excluded. Additionally, the Phenoscanner database was utilized to identify and exclude SNVs associated with confounders or the outcome [19].

### 2.4. MR Method

Prior to executing MR analyses, the F-statistic for each selected IV was calculated to assess the potential bias associated with weak IV. In every instance within this study, the F-statistic exceeded 10, signifying that the chosen SNVs were effective in minimizing bias attributable to weak IVs, and were consequently incorporated into the MR analysis [20]. The F-statistic is calculated as F = R^2^ × (*n* − 2)/(1 – R^2^), where R² represents the proportion of exposure variance explained by each IV. Specifically, R^2^ = 2 × EAF × (1 − EAF) × Beta^2^, with Beta denoting the allele effect size and EAF the frequency of the effect allele. SNVs exhibiting an F value < 10 was excluded to ensure analytical robustness and to mitigate the influence of weak IV bias.

The primary analytical technique employed was the IVW method, complemented by MR-Egger regression, WM, simple mode, and weighted mode analyses [21]. These methods were selected based on their capacitye most reliable estimates using the effective SNVs identified for the MR analysis [22]. To ascertain the validity of the findings, several diagnostic tests were conducted. Heterogeneity was evaluated using Cochran’s Q test, while MR-Egger regression served to examine the causal relationships between genetic variations and both exposure and outcome, including assessments for pleiotropy or selection bias. The MR-PRESSO test was utilized to identify outliers, providing refined estimates post-outlier-removal to enhance the accuracy and robustness of the analysis [23]. Effect sizes were expressed as odds ratios (ORs) with 95% confidence intervals.

### 2.5. Statistical Analysis

A bidirectional MR analysis was conducted to explore the causal relationship between PUFAs and PC, considering a *p*-value of less than 0.05 to be statistically significant. Heterogeneity among instrumental variables was assessed using Cochran’s Q test, erogeneity. The MR-Egger regression method assessed potential pleiotropy effects across the IVs, and directiol pleiotropy was visually represented through funnel plots. The symmetry of these plots served as an indicator of pleiotropy [23,24]. Pleiotropy bias was further addressed by identifying and removing outliers using the MR-PRESSO tool version 1.0, subsequently evaluating the impact of outlier removal on the causal inference to enhance analytical accuracy and robustness [25]. A leave-one-out sensitivity analysis was performed to verify the stability and consistency of the findings, systematically excluding each SNV to ensure result reliability [26]. Analyses were executed using the MR-PRESSO, TwoSampleMR (version 0.5.6), and Mendelian Randomization packages in R software (version 4.3.3).

## 3. Results

### 3.1. Selection of Instrumental Variables

Screening identified significant associations with:

(1) FAω3 levels (103 SNVs).

(2) DHA (83 SNVs).

(3) FAω6 levels (96 SNVs).

(4) FAω6 to FAω3 ratio (85 SNVs)..

All of the selected SNVs met the screening criteria (*p* < 0.05, R^2^ < 0.001, physical window = 10,000 kb) and were further vetted using Phenoscanner V2 to exclude any associated outcomes or confounding factors. To mitigate the effects of palindromic SNVs with medium-effect allele frequencies, alignment between exposure and outcome data led to the exclusion of specific alleles in groups 1 (rs7621223, rs2288912, rs4782568, rs5112), 3 (rs4704210, rs4766578, rs9616847), and 4 (rs7621223, rs12694696, rs12976395, rs7916868). All included SNVs exhibited an F-statistic above 10, with details provided in Tables S1–S4.

### 3.2. Mendelian Randomization Analysis

The random effects IVW method was employed as the primary analytical tool to investigate the genetic links between various PUFAs and PC risk. The analysis revealed that genetically inferred FAω3 levels were significantly associated with a reduced risk of PC (*p* = 0.01, OR [95% confidence interval (CI)] = 0.81 [0.70–0.95]). Similarly, the FAω3 derivative, DHA, was linked to a decreased PC risk (*p* = 0.004, OR [95% CI] = 0.77 [0.64–0.92]). Conversely, FAω6 levels showed no significant association with PC risk. However, an increased risk was observed with the FAω6 to FAω3 ratio (*p* = 0.02, OR [95% CI] = 1.24 [1.04–1.47]) (Table 1). An examination of 19 SNVs each for DHA, FAω3 levels, and the FAω6 to FAω3 ratio did not reveal any inverse associations (see Figures S1–S4).

### 3.3. Sensitivity Analysis

**Figure 2:**
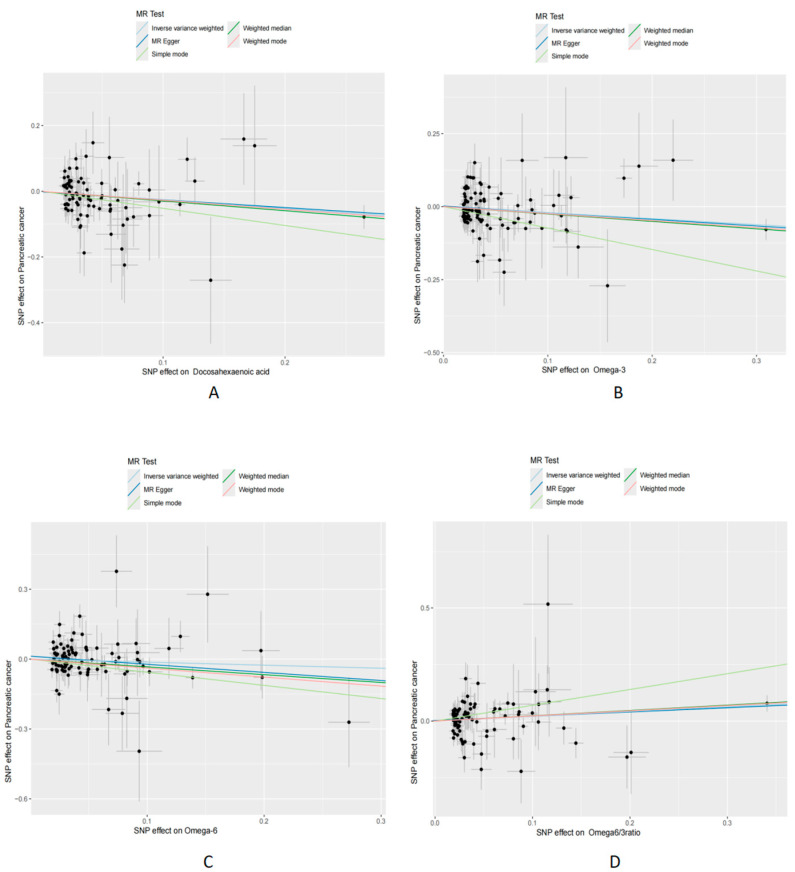
Scatterplot for MR analysis of causal effects of PUFAs on PC. (**A**) DHA on PC; (**B**) FAω3 levels on PC; (**C**) FAω6 levels on PC; (**D**) FAω6 to FAω3 ratio on PC. MR: Mendelian Randomization; PC: pancreatic cancer; FAω3 levels: Omega-3 fatty acid levels; FAω6 levels: Omega-6 fatty acid levels. FAω6 to FAω3 ratio: the ratio of Omega-6 to Omega-3.

**Figure 3:**
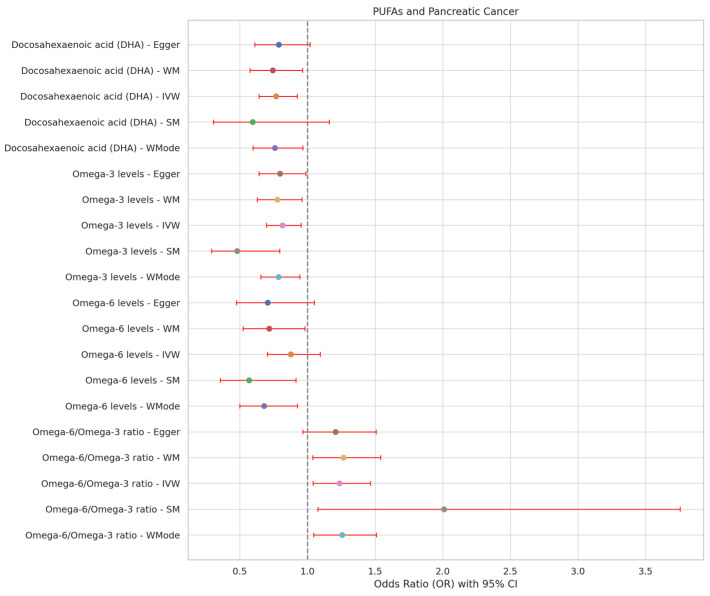
Exploring the association between PUFAs and PC using forest plots. MR: Mendelian Randomization; OR: Odds Ratio; CI: Confidence Interval; PC: pancreatic cancer.

To validate the robustness of these findings, MR-Egger regression analysis was conducted, indicating no significant evidence of directional pleiotropy or heterogeneity. Scatter plots (Figure 2, and reverse MR in Figure S4) consistently supported the causal relationships identified with the IVW, MR-Egger regression, and WM methods. Funnel plots (Figure S2, including reverse analyses) were employed to visually assess directional pleiotropy, demonstrating a symmetrical distribution. The influence of individual SNVs on the collective results was examined through leave-one-out plots (Figure S1, including reverse analyses). This approach involved sequentially excluding each SNV, recalculating the results, and observing any changes, thereby evaluating each SNP’s impact. Further MR analyses are detailed in Figure 3. These analyses collectively suggest that the MR findings are not disproportionately affected by any single SNV.

## 4. Discussion

This research utilizes a bidirectional MR approach to investigate the potential causal links between PUFAs and PC. Employing a two-sample MR methodology, we systematically analyzed how genetic variations influencing FAω3 levels, DHA, FAω6 levels, and the FAω6 to FAω3 relate to PC risk. Our results, reinforced by sensitivity analyses, indicate that genetic variants associated with increased FAω3 levels and DHA specifically correlate with a decreased PC risk. Conversely, FAω6 levels did not demonstrate a protective effect against PC. Significantly, an elevated FAω6 to FAω3 ratio was positively correlated with PC risk. Although the observed effect sizes are small, these findings have significant clinical implications. The cumulative impact of small effect sizes in large populations can be substantial. Furthermore, regulating PUFAs through dietary interventions may offer a simple and feasible strategy for the prevention of PC. The establishment of these causal associations underscores the potential for dietary management strategies not just in symptom alleviation, but also in significantly enhancing clinical outcomes and patient quality of life [27].

The role of PUFAs, particularly FAω3 and DHA, in reducing PC risk is increasingly supported by evidence [28,29]. The anti-inflammatory and antioxidant properties of PUFAs are posited as key mechanisms in cancer prevention [30,31]. Extensive observational research has highlighted the impact of PUFAs on PC risk, linking specific dietary fatty acid profiles with PC incidence. PUFAs, especially FAω3 and FAω6, are essential for physiological health and disease prevention due to their involvement in critical biological processes [13,32]. These include cell membrane composition, inflammation regulation, and signal transduction [33,34,35]. FAω3 are typically associated with anti-inflammatory effects, whereas FAω6 tend to promote inflammatory responses. This dichotomy suggests that dietary FAω6 to FAω3 ratios may crucially influence inflammatory disease risks, including PC [36]. Some studies suggest that early life dietary patterns significantly impact cancer development [37,38]. The primary risk factors include a sedentary lifestyle and poor dietary choices, characterized by excessive intake of high-calorie foods and a lack of healthy foods such as those rich in FAω3, antioxidants, and fiber. These dietary habits often lead to weight gain and obesity, which promote chronic inflammation of adipose tissue, creating a favorable microenvironment for cancer initiation and progression [39]. Additionally, studies indicate that a high-calorie diet primarily consisting of red and processed meats, carbohydrates, and high-fat foods increases the risk of PC [40]. In contrast, healthy dietary habits, characterized by the consumption of fruits and vegetables rich in fiber, PUFAs, and vitamins, can positively impact human health by reducing chronic inflammation and subsequent DNA damage [41,42]. For example, adhering to a Mediterranean diet, which includes a high intake of fresh fruits, vegetables, legumes, unrefined grains, and olive oil, as well as higher nut consumption, has been shown to be associated with a lower risk of PC [38,40]. Additionally, the intake of PUFAs and their fish sources may have a protective effect against the development of PC. These associations likely relate to healthier dietary fat sources, such as fish and nuts, which are key components of cancer-preventive dietary patterns [43]. These findings further confirm that dietary interventions may provide a new strategy for the prevention of PC.

While observational studies have illuminated the potential relationship between PUFAs and PC, they have fallen short of establishing causality. GWAS emerge as a formidable resource for deciphering the genetic underpinnings of complex diseases. Leveraging large-scale GWAS data, our investigation applies the MR technique to scrutinize the association between PUFAs and PC risk. This approach circumvents the confounding factors and reverse causation challenges inherent in traditional observational studies by utilizing genetic variations as IVs. These variations, which correlate directly with plasma fatty acid levels, are determined at birth and remain uninfluenced by external confounding factors.

PC remains a formidable global health issue, characterized by its aggressive nature and bleak prognosis. Lipid metabolism alterations, especially those involving PUFAs, play a pivotal role in PC development. FAω3, a subtype of PUFAs, can modulate cell life cycles, potentially curtailing tumor growth by promoting cancer cell apoptosis and inhibiting proliferation. This is achieved through modulation of signaling pathways, including the suppression of the nuclear factor kappa B (NF-κB) pathway and activation of the PPAR pathway, thereby influencing tumor cell life cycles [44,45]. Additionally, the antioxidant properties of FAω3 reduce oxidative stress, shielding cells from free radical damage—a critical factor in PC progression [46]. Furthermore, PUFAs anti-inflammatory properties are crucial, given inflammation’s role in pancreatic carcinogenesis. By altering inflammatory pathways and reducing pro-inflammatory cytokine production, PUFAs may lower PC risk. Moreover, through regulation of lipid metabolism and energy balance, FAω3 could indirectly mitigate PC risk by improving blood lipid profiles and enhancing insulin sensitivity [47]. Notably, statins inhibit 3-hydroxy-3-methylglutaryl coenzyme A reductase (HMG-CoA reductase), reducing cholesterol synthesis while exhibiting anti-inflammatory and antiproliferative effects. Statins can inhibit pro-inflammatory signaling pathways, such as nuclear factor kappa B (NF-κB), thereby reducing inflammatory responses and potentially inhibiting the prevention and progression of PC [48]. Studies have shown that statins can alter the lipid composition of cell membranes and affect fatty acid metabolic pathways, potentially regulating the ratio of FAω3 to FAω6 fatty acids [49]. Statins not only lower cholesterol, but also demonstrate significant anti-inflammatory and antiproliferative effects, which may aid in preventing and progressing PC [50,51]. Their ability to regulate lipid metabolism and inhibit key cellular pathways highlights their potential value as adjunctive cancer therapy. Although these findings require further experimental validation, rationally adjusting the proportion of PUFAs in the diet and applying lipid-regulating drugs such as statins may provide new strategies for preventing and treating PC, offering more robust evidence for clinical practice.

Our study offers several significant advantages. Firstly, to address the insufficient causal relationship between PUFAs and PC, we employed MR analysis. This method rigorously examines the causal relationship between PUFAs and PC by utilizing the latest large-scale GWAS data, thereby overcoming the limitations of traditional observational studies, such as reverse causality and confounding factors, and providing more precise estimates. Secondly, by carefully selecting instrumental variables for MR analysis, we significantly improved the accuracy and reliability of our results. This rigorous selection process reduces the impact of potential confounding variables, enhancing the validity of causal inference. Additionally, we incorporated various sensitivity analyses, including tests for pleiotropy and heterogeneity, to ensure the integrity and credibility of the study results. Pleiotropy analysis helps us identify and exclude instrumental variables with pleiotropic effects, preventing potential biases. Heterogeneity tests evaluate the consistency among different sub-samples, ensuring the robustness of the results. Lastly, understanding the association between PUFAs and PC risk can provide scientific evidence for dietary interventions, potentially helping to reduce PC risk. We also found that lipid-regulating drugs, such as statins, may regulate the PUFA ratio. Future considerations could include combining these drugs (e.g., statins) with dietary PUFAs, potentially producing unexpected effects on the prevention and treatment of PC. These advantages not only enhance the reliability and scientific validity of our research conclusions, but also provide valuable references for future studies.

This study faces some limitations. Although our findings have potential for significant translational value, biases and inherent challenges may exist when applying these results to clinical practice. Firstly, sample diversity and external validity are primary considerations. Our research is based on data from specific populations, and may not be applicable to all races and geographical backgrounds. To ensure the universality of the results, future studies should include more diverse participants. Secondly, lifestyle factors (such as smoking and alcohol abuse) and environmental factors may influence the association between levels of PUFAs and PC risk. Different dietary habits, environmental exposures, and socioeconomic factors can affect the results, requiring further exploration in future studies. Thirdly, our study focused only on PUFAs, excluding other important metabolic factors associated with PC. Future research should broaden the scope to include more metabolic factors, and consider additional potential confounders to improve the comprehensiveness and applicability of the study. Fourthly, according to Rubanovich and Khromov-Borisov, markers with OR < 2.2 have limitations in predictive efficiency [52]. Although the association between PUFAs and PC risk found in this study is statistically significant, the effect size is small (OR < 2.2), which may limit its clinical predictive value. Future research should use larger sample sizes and more efficient markers to improve predictive efficiency and clinical applicability. Additionally, attention should be paid to the impact of marker population frequency on the results and further exploration of the heterogeneity of these associations in different subgroups. Although thorough sensitivity analyses were conducted to test the assumptions of MR studies, completely addressing the challenges posed by pleiotropy in IVs remains difficult, potentially introducing bias. Future research needs more rigorous methods to address this issue. Finally, although our study provides strong genetic evidence for the association between PUFAs and PC, individual differences in response to dietary supplements increase the complexity of clinical applications. To address these challenges, future research should be validated in larger and more representative populations to improve the reliability and generalizability of the results and explore the feasibility of dietary interventions to regulate PUFAs for the prevention and treatment of PC. Moreover, in the study of clinical tumor single-cell bioinformatics and autoimmune disease mouse experiments, bioinformatics analysis is widely used to validate and support experimental results [53,54]. Similarly, future research can use bioinformatics tools to conduct in-depth analyses of gene expression, gene variations, and biomarkers, screen potential drug molecules through computer simulations, and explore the application of bioinformatics to cancer treatment targets. This approach can not only verify the association between PUFAs and PC risk, but also provide a deeper mechanistic understanding, important clues for further experimental research, and crucial evidence for the development of new prevention and treatment strategies.

## 5. Conclusions

Our research provides robust genetic evidence that suggests a potential causal relationship between PUFAs and PC risk. Specifically, higher levels of FAω3 and DHA are associated with a lower risk of PC, while an increased ratio of FAω6 to FAω3 appears to elevate this risk. These findings imply that dietary adjustments to optimize the FAω6 to FAω3 ratio may significantly reduce the incidence of PC. Our study emphasizes the important role of these fatty acids in PC prevention. The protective effect of higher FAω3 and DHA levels suggests that increasing the intake of FAω3-rich foods (such as fish oil and flaxseeds) may be an effective strategy for reducing PC risk. Conversely, reducing the intake of FAω6-rich oils (such as corn oil and soybean oil) may also be beneficial. Furthermore, although our results strongly suggest a causal relationship, they highlight the necessity for further prospective controlled experiments (both in vitro and in vivo) to establish the exact mechanisms underlying this association. Understanding how FAω3 and FAω6 influence cellular processes related to inflammation, cell proliferation, and apoptosis is crucial for developing targeted prevention and treatment strategies. Therefore, we advocate for more in-depth MR analyses in future studies to elucidate the roles and long-term impacts of PUFAs, particularly FAω3 and FAω6, in PC prevention and treatment. Assessing their effects on treatment outcomes and patient survival rates is also essential. By deepening our understanding of FAω3 and FAω6, as well as how they regulate cancer risk in combination with lipid-lowering drugs (statins), we may develop new dietary and treatment strategies to reduce the burden of PC.

## Figures and Tables

**Table 1:** Assessing the causal effects of PUFAs on the risk of PC.

Exposure	Outcome	Method	nsnp	pval	or	or_lci 95	or_uci 95
Docosahexaenoic acid	Pancreatic cancer	MR Egger	83	0.074	0.79	0.61	1.02
Docosahexaenoic acid	Pancreatic cancer	Weighted median	83	0.03	0.74	0.57	0.96
Docosahexaenoic acid	Pancreatic cancer	Ivw	83	0.004	0.77	0.64	0.92
Docosahexaenoic acid	Pancreatic cancer	Simple mode	83	0.13	0.59	0.30	1.16
Docosahexaenoic acid	Pancreatic cancer	Weighted mode	83	0.03	0.76	0.60	0.97
Omega-3 levels	Pancreatic cancer	MR Egger	103	0.06	0.80	0.64	0.99
Omega-3 levels	Pancreatic cancer	Weighted median	103	0.02	0.78	0.63	0.96
Omega-3 levels	Pancreatic cancer	Ivw	103	0.01	0.81	0.70	0.95
Omega-3 levels	Pancreatic cancer	Simple mode	103	0.005	0.48	0.29	0.79
Omega-3 levels	Pancreatic cancer	Weighted mode	103	0.01	0.79	0.66	0.94
Omega-6 levels	Pancreatic cancer	MR Egger	96	0.09	0.71	0.47	1.05
Omega-6 levels	Pancreatic cancer	Weighted median	96	0.04	0.72	0.52	0.98
Omega-6 levels	Pancreatic cancer	Ivw	96	0.25	0.88	0.70	1.09
Omega-6 levels	Pancreatic cancer	Simple mode	96	0.02	0.57	0.35	0.91
Omega-6 levels	Pancreatic cancer	Weighted mode	96	0.02	0.68	0.50	0.93
Omega-6/-3 ratio	Pancreatic cancer	MR Egger	85	0.10	1.21	0.97	1.51
Omega-6/-3 ratio	Pancreatic cancer	Weighted median	85	0.02	1.27	1.04	1.54
Omega-6/-3 ratio	Pancreatic cancer	Ivw	85	0.02	1.24	1.04	1.47
Omega-6/-3 ratio	Pancreatic cancer	Simple mode	85	0.03	2.01	1.08	3.75
Omega-6/-3 ratio	Pancreatic cancer	Weighted mode	85	0.02	1.26	1.04	1.51

This Table, employing IVW as the primary method, demonstrates that FAω3 and DHA serve as protective factors against PC, whereas the FAω6 to FAω3 ratio constitutes a risk factor for PC.

## Data Availability

The data supporting the findings of this study are available from the UK Biobank. Requests for access to these data should be directed to the corresponding author.

## Supplementary Materials

The following supporting information can be downloaded at: https://www.mdpi.com/article/10.3390/cimb46060360/s1, Supplementary Figure S1: Leave-one-out sensitivity MR analysis test of PUFA and PC; Supplementary Figure S2: MR Funnel plot of analysis of PUFA and PC; Supplementary Figure S3: Leave-one-out sensitivity of reverse MR; Supplementary Figure S4: Scatter plot of reverse MR; Supplementary Tables S1–S4: DHA, Omega3, Omega6, Omega6 to Omega3 ratio each F value of SNV.
