# Supplementary material for: Employing Bidirectional Two-Sample Mendelian Randomization Analysis to Verify the Potential of Polyunsaturated Fatty Acid Levels in the Prevention of Pancreatic Cancer
Source: Curr Issues Mol Biol. 2024 Jun 14;46(6):6041–51. doi: 10.3390/cimb46060360 (PMC11202278; doi:10.3390/cimb46060360)
Supplement: Supplementary file 1 [file cimb-46-00360-s001.zip › Supplemental Figures.pdf]

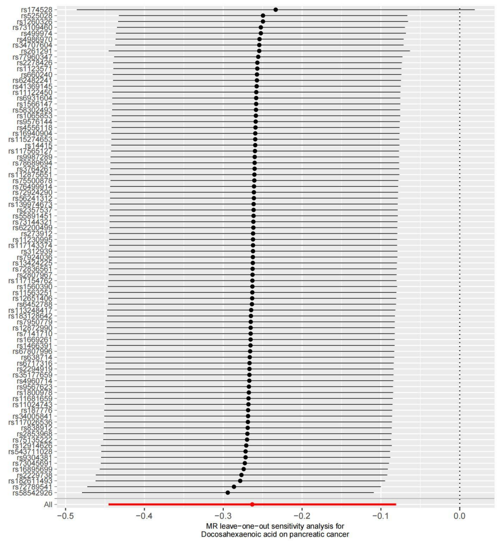
MR leave−one−out sensitivity analysis for
Docosahexaenoic acid on pancreatic cancer
All

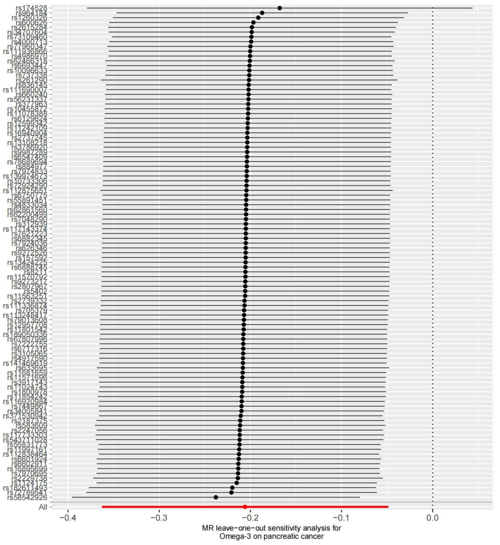
MR leave−one−out sensitivity analysis for
Omega−3 on pancreatic cancer
All

A

B

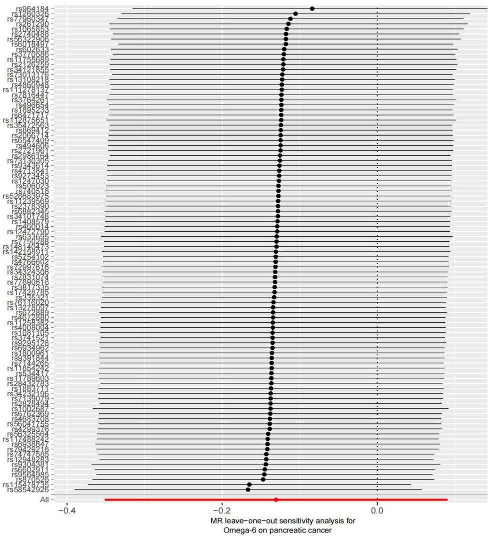
MR leave−one−out sensitivity analysis for
Omega−6 on pancreatic cancer
All

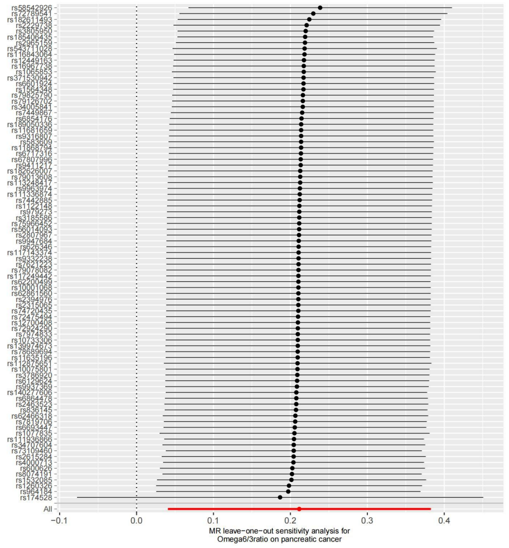
MR leave−one−out sensitivity analysis for
Omega6/3ratio on pancreatic cancer
All

C

D

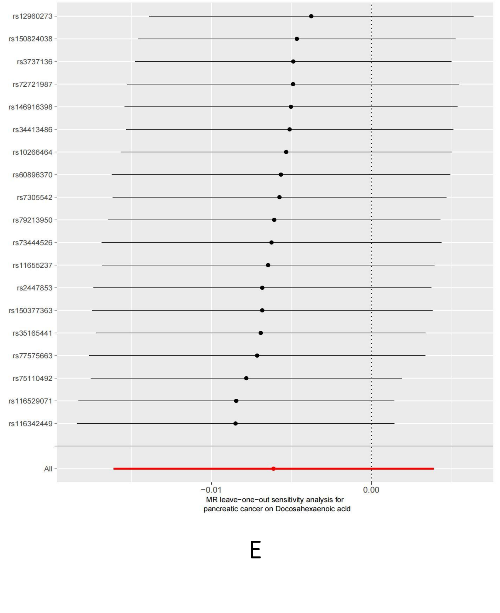
rs12960273
rs150824038
rs3737136
rs72721987
rs146916398
rs34413486
rs10266464
rs60896370
rs7305542
rs79213950
rs73444526
rs11655237
rs2447853
rs150377363
rs35165441
rs77575663
rs75110492
rs116529071
rs116342449
All
−0.01
0.00
MR leave−one−out sensitivity analysis for
pancreatic cancer on Docosahexaenoic acid

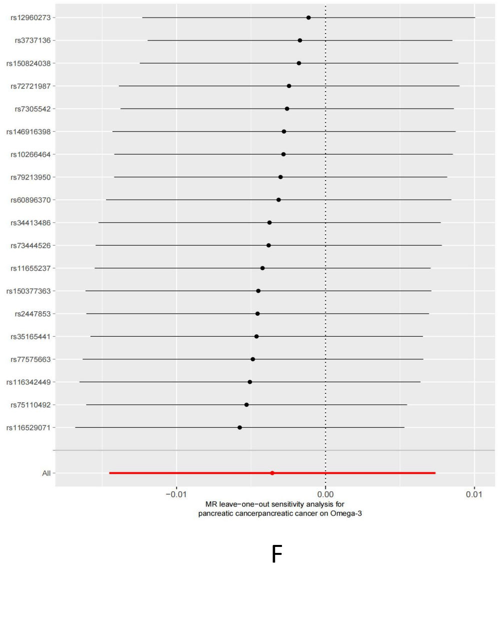
rs12960273
rs3737136
rs150824038
rs72721987
rs7305542
rs146916398
rs10266464
rs79213950
rs60896370
rs34413486
rs73444526
rs11655237
rs150377363
rs2447853
rs35165441
rs77575663
rs116342449
rs75110492
rs116529071
All
−0.01
0.00
0.01
MR leave−one−out sensitivity analysis for
pancreatic cancerpancreatic cancer on Omega−3

E

F

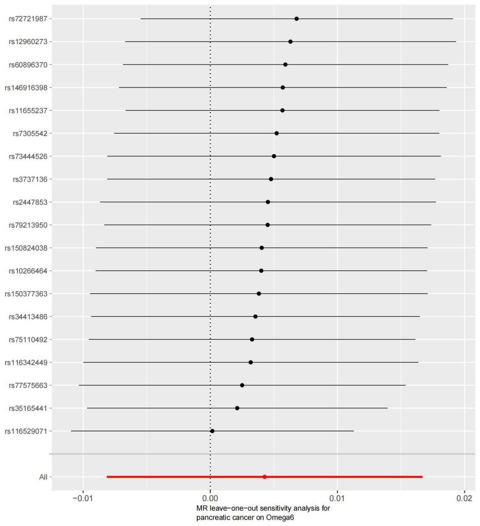

G

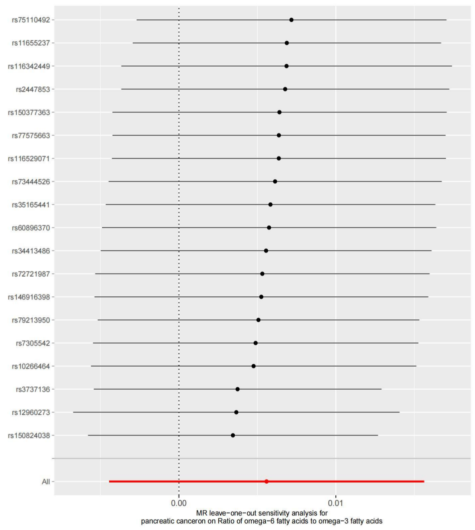

H

Supplementary Figure S1

Leave one out sensitivity tests of MR analyses of PUFAs and PC. Calculate the MR results of the remaining IVs after removing the IVs one by one. A: Docosahexaenoic acid on pancreatic cancer; B: Omega3 levels on pancreatic cancer; C: Omega6 levels on pancreatic cancer; D: Omega6/3 on pancreatic cancer E: pancreatic cancer on Docosahexaenoic acid; F: Pancreatic cancer on Omega3 levels; G: pancreatic cancer on Omega6 levels; H: Pancreatic cancer on Omega 6/3ratio

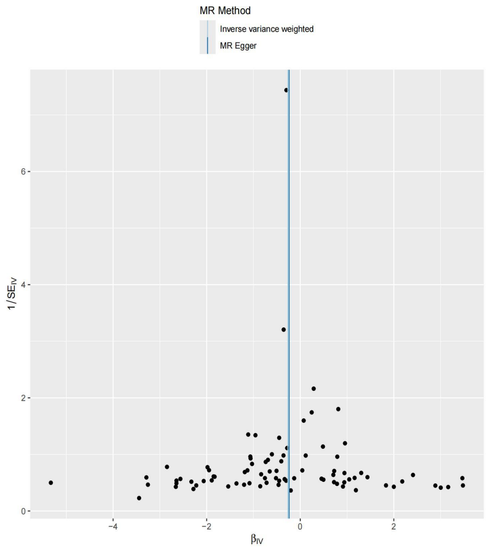

A

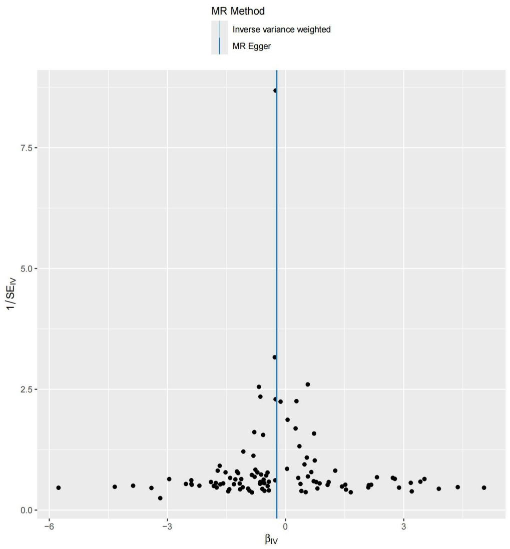

B

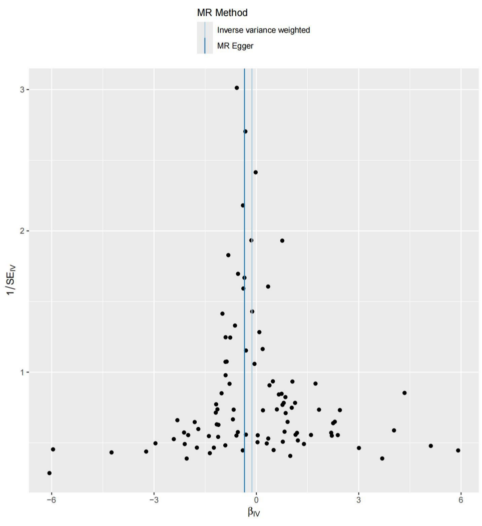

C

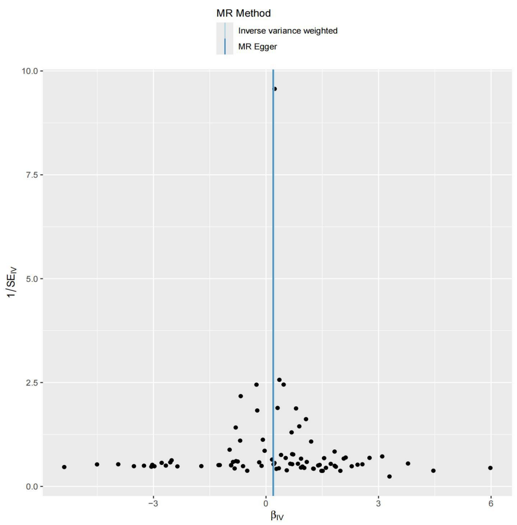

D

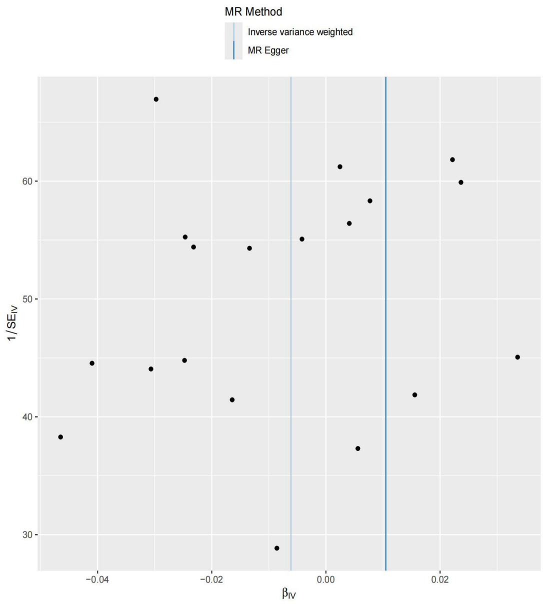

E

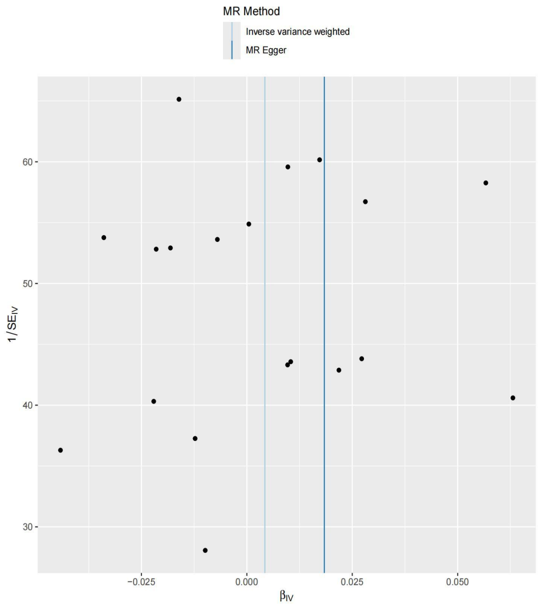

F

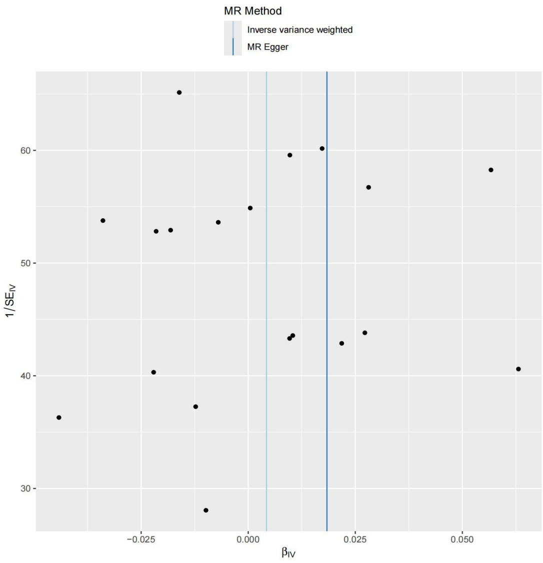

G

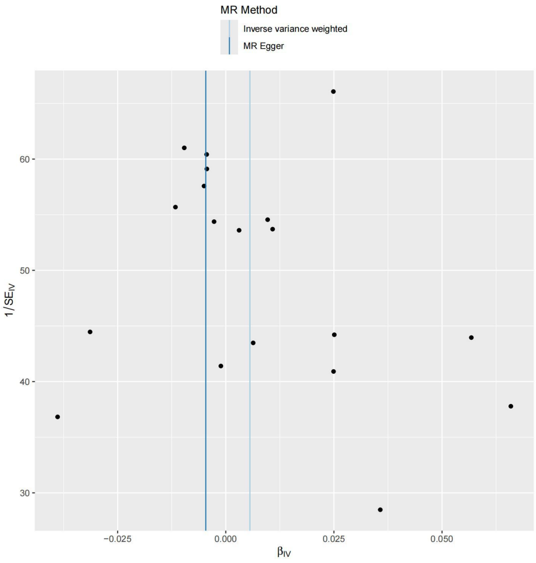

H

Supplementary Figure S2.

Funnel plot of MR analyses of PUFAs and PC . A: Docosahexaenoic acid on pancreatic cancer; B: Omega3 levels on pancreatic cancer; C: Omega6 levels on pancreatic cancer; D: Omega6/3 on pancreatic cancer E: pancreatic cancer on docosahexaenoic acid; F: Pancreatic cancer on Omega3 levels; G: pancreatic cancer on Omega6 levels; H: Pancreatic cancer on Omega 6/3ratio

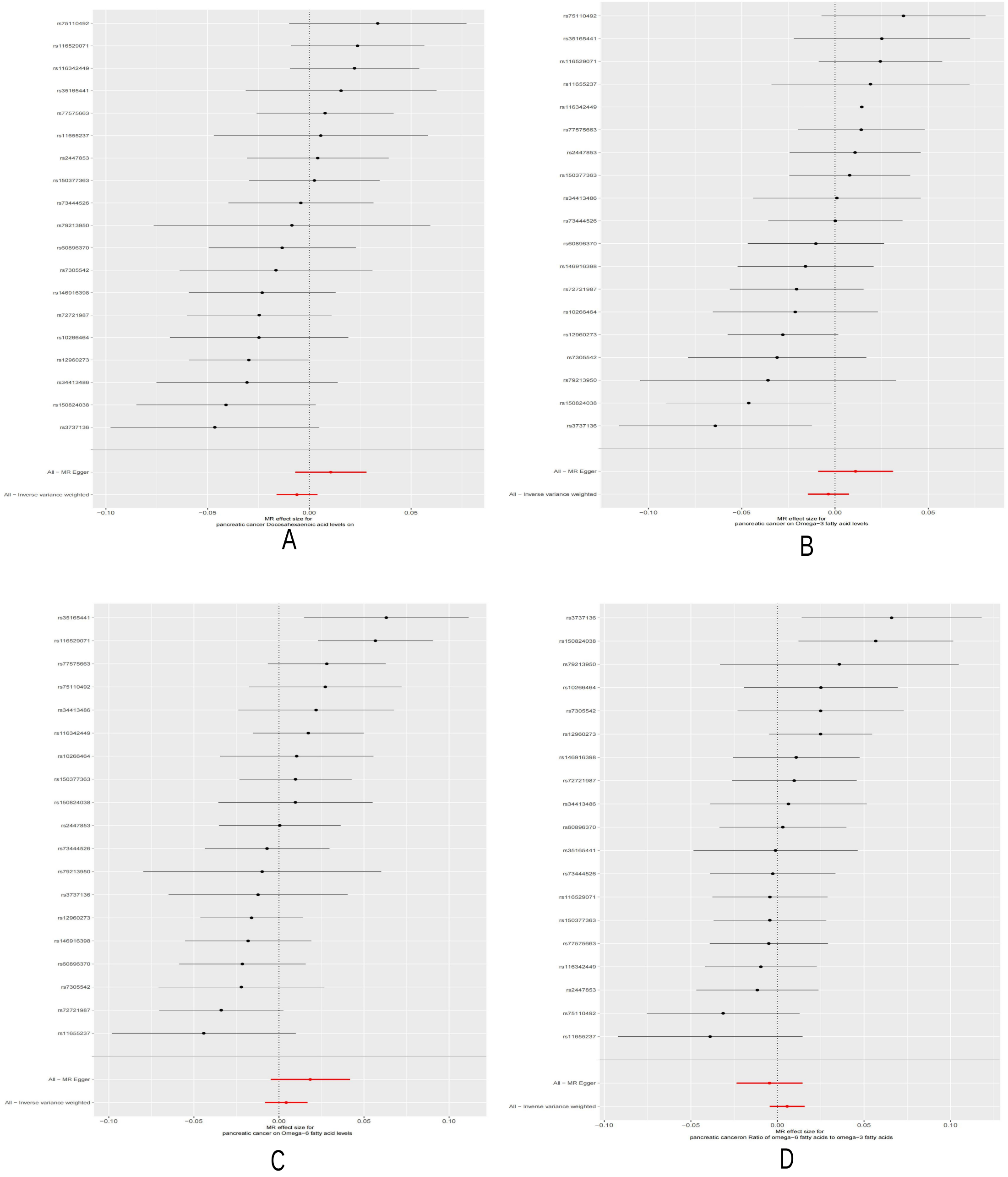

Supplementary Figure S3（Reverse MR）.

MR analysis of forest plots of PUFAs and PCs. A: pancreatic cancer on Docosahexaenoic acid; B: Pancreatic cancer on Omega3 levels;C: pancreatic cancer on Omega6 levels; D: Pancreatic cancer on Omega 6/3ratio

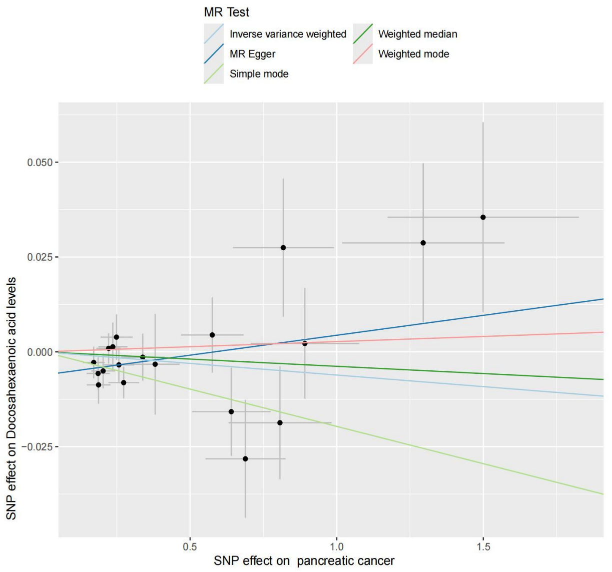

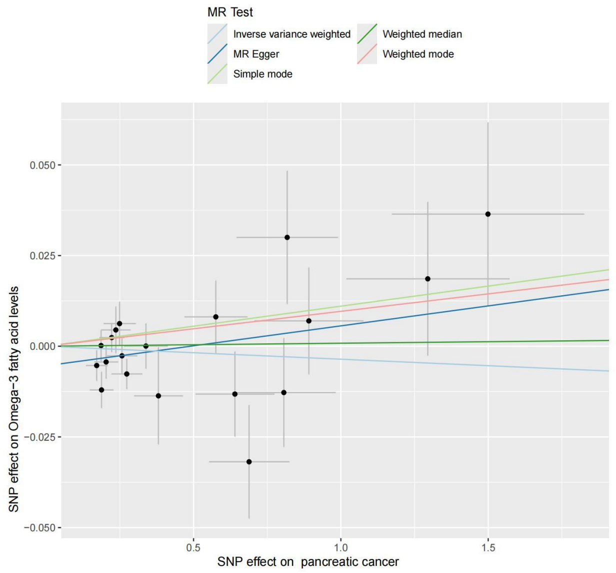

A

B

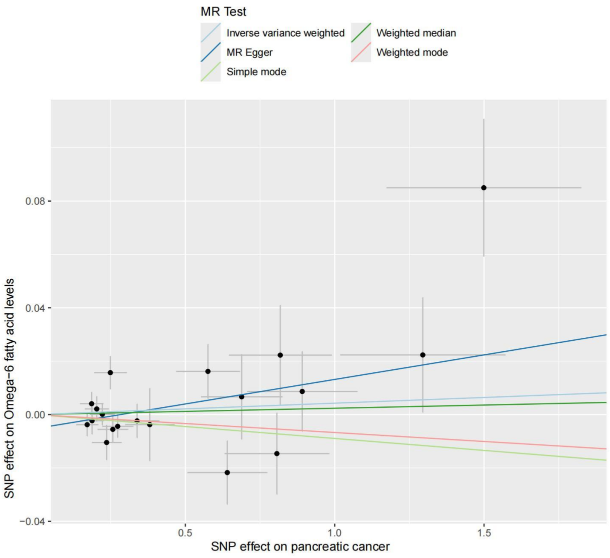

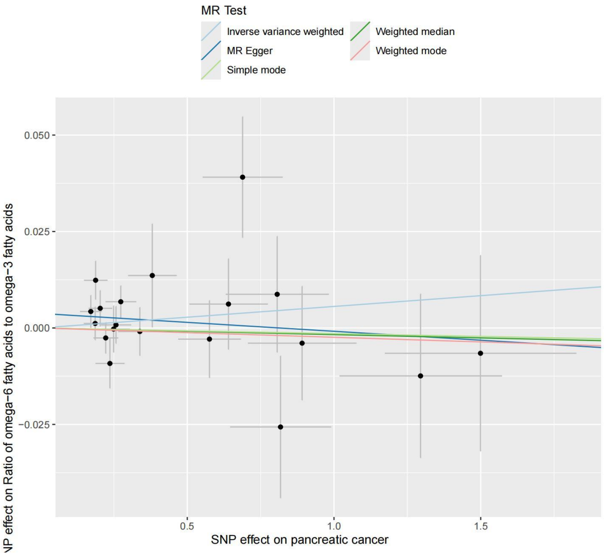

C

D

Supplementary Figure S4（Reverse MR）. Scatterplot for MR analysis of causal effects of PC on PUFA. A: pancreatic cancer on docosahexaenoic acid; B: Pancreatic cancer on Omega3 levels;C: pancreatic cancer on Omega6 levels; D: Pancreatic cancer on Omega 6/3ratio
